# Amiloride hydro­chloride methanol disolvate

**DOI:** 10.1107/S1600536810017484

**Published:** 2010-05-15

**Authors:** Cuong Quoc Ton, Michael Bolte

**Affiliations:** a Institut für Organische Chemie, J. W. Goethe-Universität Frankfurt, Max-von-Laue-Strasse 7, 60438 Frankfurt/Main, Germany; b Institut für Anorganische Chemie, J. W. Goethe-Universität Frankfurt, Max-von-Laue-Strasse 7, 60438 Frankfurt/Main, Germany

## Abstract

In the crystal of the title compound [systematic name: 2-(3,5-diamino-6-chloro­pyrazin-2-ylcarbon­yl)guanidinium chloride methanol disolvate], C_6_H_9_ClN_7_O^+^·Cl^−^·2CH_3_OH , the components are connected by N—H⋯N, N—H⋯Cl, N—H⋯O, O—H⋯Cl and O—H⋯O hydrogen bonds into a three-dimensional network. The dihedral angle between the aromatic ring and the guanidine residue is 6.0 (2)°.

## Related literature

For other salts  of amiloride, see: Pretscher *et al.* (2001 ▶); Zeslawska *et al.* (2004 ▶).
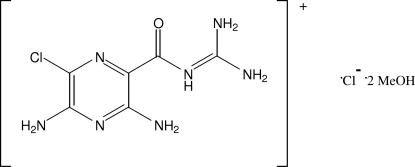


## Experimental

### 

#### Crystal data

C_6_H_9_ClN_7_O^+^·Cl^−^·2CH_4_O
*M* *_r_* = 330.19
Monoclinic, 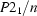

*a* = 5.9473 (5) Å
*b* = 16.7278 (17) Å
*c* = 14.7784 (15) Å
β = 90.080 (8)°
*V* = 1470.2 (2) Å^3^
*Z* = 4
Mo *K*α radiation
μ = 0.46 mm^−1^
*T* = 173 K
0.30 × 0.25 × 0.20 mm

#### Data collection

Stoe IPDS II two-circle diffractometer
Absorption correction: multi-scan (*MULABS*; Spek, 2009 ▶; Blessing, 1995 ▶) *T*_min_ = 0.874, *T*_max_ = 0.914
19184 measured reflections
2739 independent reflections
1852 reflections with *I* > 2σ(*I*)
*R*_int_ = 0.071

#### Refinement

*R*[*F* ^2^ > 2σ(*F* ^2^)] = 0.050
*wR*(*F* ^2^) = 0.080
*S* = 0.96
2739 reflections
212 parameters
9 restraints
H atoms treated by a mixture of independent and constrained refinement
Δρ_max_ = 0.22 e Å^−3^
Δρ_min_ = −0.25 e Å^−3^

### 

Data collection: *X-AREA* (Stoe & Cie, 2001 ▶); cell refinement: *X-AREA*; data reduction: *X-AREA*; program(s) used to solve structure: *SHELXS97* (Sheldrick, 2008 ▶); program(s) used to refine structure: *SHELXL97* (Sheldrick, 2008 ▶); molecular graphics: *XP* (Sheldrick, 2008 ▶); software used to prepare material for publication: *SHELXL97*.

## Supplementary Material

Crystal structure: contains datablocks I, global. DOI: 10.1107/S1600536810017484/ng2772sup1.cif

Structure factors: contains datablocks I. DOI: 10.1107/S1600536810017484/ng2772Isup2.hkl

Additional supplementary materials:  crystallographic information; 3D view; checkCIF report

## Figures and Tables

**Table 1:** Hydrogen-bond geometry (Å, °)

*D*—H⋯*A*	*D*—H	H⋯*A*	*D*⋯*A*	*D*—H⋯*A*
N31—H31*A*⋯N4^i^	0.88 (1)	2.14 (1)	2.996 (3)	165 (3)
N31—H31*B*⋯Cl1^ii^	0.87 (1)	2.50 (2)	3.281 (3)	150 (3)
N51—H51*A*⋯Cl1^iii^	0.88 (1)	2.54 (1)	3.396 (3)	165 (3)
N51—H51*B*⋯O11	0.88 (1)	2.11 (2)	2.781 (3)	133 (3)
N12—H12⋯O2*M*^iv^	0.87 (1)	2.14 (2)	2.912 (3)	149 (3)
N14—H14*A*⋯Cl1^v^	0.88 (1)	2.34 (1)	3.188 (3)	162 (3)
N14—H14*B*⋯O2*M*^iv^	0.88 (1)	1.93 (2)	2.783 (3)	162 (3)
N15—H15*A*⋯Cl1^v^	0.88 (1)	2.61 (2)	3.367 (3)	145 (3)
N15—H15*B*⋯O11	0.88 (1)	2.03 (3)	2.688 (3)	131 (3)
O1*M*—H1*M*⋯Cl1	0.84	2.26	3.091 (2)	171
O2*M*—H2*M*⋯O1*M*	0.84	1.91	2.745 (3)	170

Symmetry codes: (i) 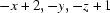; (ii) 
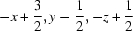
; (iii) 
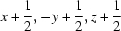
; (iv) 
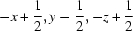
; (v) 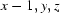.

**Fig. 1.**
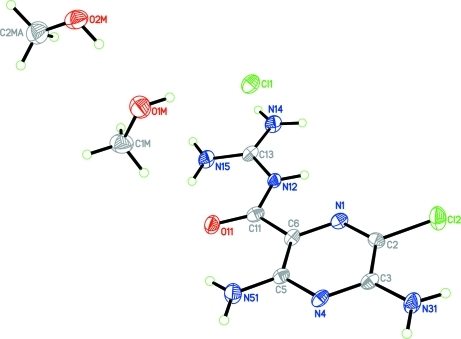
Perspective view of the asymmetric unit of the title compound. Displacement ellipsoids are at the 50° probability level.

## supplementary crystallographic information

### Experimental

Crystals of the title structure were obtained by recrystallization of amiloride hydrochloride (100 mg) from a methanol (3 g) solution.

### Refinement

H atoms bonded to O and C were geometrically positioned and refined using a riding model with fixed individual displacement parameters [U(H) = 1.5 U_eq_(C,*O*)] using a riding model with C—H = 0.98Å and O—H = 0.84 Å, respectively. The methyl and hydroxyl groups were allowed to rotate but not to tip. H atoms bonded to N were refined with a distance restraint of 0.88 (1)Å and with U(H) = 1.2 U_eq_(N).

### Crystal data

**Table:**

C_6_H_9_ClN_7_O^+^·Cl^−^·2CH_4_O	*F*(000) = 688
*M**_r_* = 330.19	*D*_x_ = 1.492 Mg m^−^^3^
Monoclinic, *P*2_1_/*n*	Mo *K*α radiation, λ = 0.71073 Å
Hall symbol: -P 2yn	Cell parameters from 11166 reflections
*a* = 5.9473 (5) Å	θ = 3.7–25.7°
*b* = 16.7278 (17) Å	µ = 0.46 mm^−^^1^
*c* = 14.7784 (15) Å	*T* = 173 K
β = 90.080 (8)°	Block, colourless
*V* = 1470.2 (2) Å^3^	0.30 × 0.25 × 0.20 mm
*Z* = 4	

### Data collection

**Table:**

Stoe IPDS II two-circle diffractometer	2739 independent reflections
Radiation source: fine-focus sealed tube	1852 reflections with *I* > 2σ(*I*)
graphite	*R*_int_ = 0.071
ω scans	θ_max_ = 25.6°, θ_min_ = 3.6°
Absorption correction: multi-scan (*MULABS*; Spek, 2009; Blessing, 1995)	*h* = −7→7
*T*_min_ = 0.874, *T*_max_ = 0.914	*k* = −20→20
19184 measured reflections	*l* = −17→17

### Refinement

**Table:**

Refinement on *F*^2^	Primary atom site location: structure-invariant direct methods
Least-squares matrix: full	Secondary atom site location: difference Fourier map
*R*[*F*^2^ > 2σ(*F*^2^)] = 0.050	Hydrogen site location: inferred from neighbouring sites
*wR*(*F*^2^) = 0.080	H atoms treated by a mixture of independent and constrained refinement
*S* = 0.96	*w* = 1/[σ^2^(*F*_o_^2^) + (0.0253*P*)^2^] where *P* = (*F*_o_^2^ + 2*F*_c_^2^)/3
2739 reflections	(Δ/σ)_max_ < 0.001
212 parameters	Δρ_max_ = 0.22 e Å^−^^3^
9 restraints	Δρ_min_ = −0.25 e Å^−^^3^

### Special details

**Table:**

Geometry. All esds (except the esd in the dihedral angle between two l.s. planes) are estimated using the full covariance matrix. The cell esds are taken into account individually in the estimation of esds in distances, angles and torsion angles; correlations between esds in cell parameters are only used when they are defined by crystal symmetry. An approximate (isotropic) treatment of cell esds is used for estimating esds involving l.s. planes.
Refinement. Refinement of *F*^2^ against ALL reflections. The weighted *R*-factor wR and goodness of fit *S* are based on *F*^2^, conventional *R*-factors *R* are based on *F*, with *F* set to zero for negative *F*^2^. The threshold expression of *F*^2^ > σ(*F*^2^) is used only for calculating *R*-factors(gt) etc. and is not relevant to the choice of reflections for refinement. *R*-factors based on *F*^2^ are statistically about twice as large as those based on *F*, and *R*- factors based on ALL data will be even larger.

### Fractional atomic coordinates and isotropic or equivalent isotropic displacement parameters (Å^2^)

**Table:**

	*x*	*y*	*z*	*U*_iso_*/*U*_eq_	
N1	0.4136 (4)	0.07364 (14)	0.32999 (16)	0.0187 (6)	
C2	0.5851 (5)	0.02607 (17)	0.32413 (18)	0.0179 (6)	
Cl2	0.62266 (14)	−0.02883 (5)	0.22493 (5)	0.0315 (2)	
C3	0.7485 (5)	0.01732 (16)	0.39568 (19)	0.0182 (6)	
N31	0.9232 (4)	−0.03176 (16)	0.38692 (17)	0.0241 (6)	
H31A	1.006 (5)	−0.0400 (18)	0.4354 (14)	0.029*	
H31B	0.939 (5)	−0.0621 (16)	0.3394 (14)	0.029*	
N4	0.7256 (4)	0.06106 (13)	0.47133 (16)	0.0179 (5)	
C5	0.5473 (5)	0.11111 (16)	0.47856 (19)	0.0173 (6)	
N51	0.5303 (5)	0.15414 (15)	0.55477 (18)	0.0256 (6)	
H51A	0.624 (4)	0.1497 (18)	0.6007 (15)	0.031*	
H51B	0.410 (3)	0.1839 (16)	0.561 (2)	0.031*	
C6	0.3874 (5)	0.11725 (17)	0.40695 (19)	0.0168 (6)	
C11	0.1930 (5)	0.17089 (18)	0.4093 (2)	0.0202 (7)	
O11	0.1433 (4)	0.21444 (12)	0.47391 (14)	0.0249 (5)	
N12	0.0642 (4)	0.16832 (14)	0.33015 (15)	0.0173 (5)	
H12	0.110 (5)	0.1370 (14)	0.2874 (15)	0.021*	
C13	−0.1158 (5)	0.21632 (17)	0.3097 (2)	0.0196 (7)	
N14	−0.2054 (4)	0.20867 (16)	0.22820 (18)	0.0250 (6)	
H14A	−0.326 (3)	0.2377 (16)	0.218 (2)	0.030*	
H14B	−0.134 (5)	0.1739 (15)	0.1935 (18)	0.030*	
N15	−0.1985 (4)	0.26602 (16)	0.37008 (18)	0.0250 (6)	
H15A	−0.310 (4)	0.2979 (16)	0.355 (2)	0.030*	
H15B	−0.141 (5)	0.2664 (19)	0.4246 (11)	0.030*	
Cl1	0.36907 (12)	0.32420 (4)	0.23835 (5)	0.02329 (18)	
O1M	0.4195 (4)	0.44145 (13)	0.39875 (15)	0.0335 (6)	
H1M	0.4001	0.4139	0.3519	0.050*	
C1M	0.2507 (6)	0.4216 (2)	0.4641 (2)	0.0355 (8)	
H1M1	0.2637	0.3650	0.4802	0.053*	
H1M2	0.1015	0.4318	0.4384	0.053*	
H1M3	0.2716	0.4545	0.5183	0.053*	
O2M	0.4108 (4)	0.59993 (13)	0.35076 (15)	0.0315 (6)	
H2M	0.4196	0.5504	0.3592	0.047*	
C2MA	0.2302 (6)	0.6318 (2)	0.4037 (2)	0.0370 (9)	
H2M1	0.0883	0.6071	0.3848	0.056*	
H2M2	0.2214	0.6898	0.3945	0.056*	
H2M3	0.2573	0.6205	0.4678	0.056*	

### Atomic displacement parameters (Å^2^)

**Table:**

	*U*^11^	*U*^22^	*U*^33^	*U*^12^	*U*^13^	*U*^23^
N1	0.0148 (14)	0.0184 (13)	0.0229 (14)	−0.0003 (11)	−0.0025 (10)	0.0018 (11)
C2	0.0171 (15)	0.0209 (15)	0.0157 (14)	−0.0018 (13)	−0.0030 (12)	0.0000 (12)
Cl2	0.0278 (5)	0.0391 (5)	0.0276 (4)	0.0122 (4)	−0.0061 (3)	−0.0114 (4)
C3	0.0142 (15)	0.0149 (15)	0.0254 (16)	−0.0011 (13)	0.0002 (12)	0.0037 (13)
N31	0.0209 (14)	0.0244 (15)	0.0268 (15)	0.0082 (12)	−0.0059 (12)	−0.0050 (12)
N4	0.0133 (13)	0.0146 (12)	0.0258 (14)	0.0018 (10)	−0.0036 (10)	0.0012 (10)
C5	0.0172 (16)	0.0129 (14)	0.0220 (16)	−0.0037 (12)	−0.0017 (12)	0.0012 (12)
N51	0.0250 (16)	0.0269 (15)	0.0250 (15)	0.0067 (12)	−0.0070 (12)	−0.0061 (12)
C6	0.0125 (15)	0.0141 (14)	0.0238 (16)	−0.0012 (12)	−0.0014 (12)	−0.0002 (12)
C11	0.0186 (16)	0.0162 (15)	0.0257 (16)	−0.0046 (13)	−0.0006 (13)	0.0037 (14)
O11	0.0265 (12)	0.0212 (11)	0.0268 (12)	0.0062 (9)	−0.0021 (9)	−0.0042 (9)
N12	0.0174 (13)	0.0166 (13)	0.0180 (13)	0.0043 (11)	−0.0024 (10)	−0.0011 (10)
C13	0.0141 (15)	0.0156 (15)	0.0290 (17)	−0.0004 (12)	0.0001 (13)	0.0027 (13)
N14	0.0197 (15)	0.0272 (15)	0.0280 (16)	0.0083 (11)	−0.0061 (12)	0.0000 (12)
N15	0.0197 (15)	0.0263 (15)	0.0290 (15)	0.0103 (12)	−0.0039 (12)	−0.0018 (12)
Cl1	0.0196 (4)	0.0206 (4)	0.0296 (4)	0.0024 (3)	−0.0022 (3)	0.0006 (3)
O1M	0.0372 (14)	0.0317 (14)	0.0317 (13)	−0.0091 (11)	0.0034 (11)	−0.0026 (10)
C1M	0.040 (2)	0.0347 (19)	0.0318 (19)	−0.0092 (17)	−0.0015 (16)	0.0054 (16)
O2M	0.0344 (14)	0.0271 (13)	0.0328 (13)	−0.0055 (11)	−0.0006 (11)	0.0064 (11)
C2MA	0.039 (2)	0.033 (2)	0.039 (2)	−0.0027 (17)	0.0032 (17)	0.0001 (16)

### Geometric parameters (Å, °)

**Table:**

N1—C2	1.297 (4)	N12—H12	0.866 (10)
N1—C6	1.360 (4)	C13—N15	1.316 (4)
C2—C3	1.443 (4)	C13—N14	1.322 (4)
C2—Cl2	1.745 (3)	N14—H14A	0.880 (10)
C3—N31	1.331 (4)	N14—H14B	0.884 (10)
C3—N4	1.343 (4)	N15—H15A	0.880 (10)
N31—H31A	0.880 (10)	N15—H15B	0.876 (10)
N31—H31B	0.872 (10)	O1M—C1M	1.432 (4)
N4—C5	1.355 (4)	O1M—H1M	0.8400
C5—N51	1.341 (4)	C1M—H1M1	0.9800
C5—C6	1.426 (4)	C1M—H1M2	0.9800
N51—H51A	0.879 (10)	C1M—H1M3	0.9800
N51—H51B	0.878 (10)	O2M—C2MA	1.433 (4)
C6—C11	1.464 (4)	O2M—H2M	0.8400
C11—O11	1.238 (3)	C2MA—H2M1	0.9800
C11—N12	1.398 (4)	C2MA—H2M2	0.9800
N12—C13	1.371 (4)	C2MA—H2M3	0.9800
			
C2—N1—C6	118.4 (2)	C11—N12—H12	117 (2)
N1—C2—C3	122.9 (3)	N15—C13—N14	121.9 (3)
N1—C2—Cl2	118.7 (2)	N15—C13—N12	120.8 (3)
C3—C2—Cl2	118.4 (2)	N14—C13—N12	117.2 (3)
N31—C3—N4	119.8 (3)	C13—N14—H14A	116 (2)
N31—C3—C2	121.1 (3)	C13—N14—H14B	114 (2)
N4—C3—C2	119.1 (3)	H14A—N14—H14B	131 (3)
C3—N31—H31A	117 (2)	C13—N15—H15A	119 (2)
C3—N31—H31B	122 (2)	C13—N15—H15B	119 (2)
H31A—N31—H31B	120 (3)	H15A—N15—H15B	122 (3)
C3—N4—C5	118.8 (2)	C1M—O1M—H1M	109.5
N51—C5—N4	117.2 (3)	O1M—C1M—H1M1	109.5
N51—C5—C6	122.3 (3)	O1M—C1M—H1M2	109.5
N4—C5—C6	120.5 (3)	H1M1—C1M—H1M2	109.5
C5—N51—H51A	124 (2)	O1M—C1M—H1M3	109.5
C5—N51—H51B	117 (2)	H1M1—C1M—H1M3	109.5
H51A—N51—H51B	119 (3)	H1M2—C1M—H1M3	109.5
N1—C6—C5	120.3 (3)	C2MA—O2M—H2M	109.5
N1—C6—C11	116.1 (2)	O2M—C2MA—H2M1	109.5
C5—C6—C11	123.6 (3)	O2M—C2MA—H2M2	109.5
O11—C11—N12	122.2 (3)	H2M1—C2MA—H2M2	109.5
O11—C11—C6	124.6 (3)	O2M—C2MA—H2M3	109.5
N12—C11—C6	113.2 (3)	H2M1—C2MA—H2M3	109.5
C13—N12—C11	126.4 (2)	H2M2—C2MA—H2M3	109.5
C13—N12—H12	116 (2)		
			
C6—N1—C2—C3	0.1 (4)	N51—C5—C6—N1	178.7 (3)
C6—N1—C2—Cl2	−178.9 (2)	N4—C5—C6—N1	−0.8 (4)
N1—C2—C3—N31	−179.6 (3)	N51—C5—C6—C11	0.6 (4)
Cl2—C2—C3—N31	−0.5 (4)	N4—C5—C6—C11	−178.9 (3)
N1—C2—C3—N4	−1.1 (4)	N1—C6—C11—O11	179.6 (3)
Cl2—C2—C3—N4	178.0 (2)	C5—C6—C11—O11	−2.2 (4)
N31—C3—N4—C5	179.5 (3)	N1—C6—C11—N12	−0.1 (4)
C2—C3—N4—C5	1.1 (4)	C5—C6—C11—N12	178.1 (3)
C3—N4—C5—N51	−179.7 (2)	O11—C11—N12—C13	6.0 (4)
C3—N4—C5—C6	−0.2 (4)	C6—C11—N12—C13	−174.3 (3)
C2—N1—C6—C5	0.8 (4)	C11—N12—C13—N15	−6.9 (4)
C2—N1—C6—C11	179.0 (3)	C11—N12—C13—N14	175.0 (3)

### Hydrogen-bond geometry (Å, °)

**Table:**

*D*—H···*A*	*D*—H	H···*A*	*D*···*A*	*D*—H···*A*
N31—H31A···N4^i^	0.88 (1)	2.14 (1)	2.996 (3)	165 (3)
N31—H31B···Cl1^ii^	0.87 (1)	2.50 (2)	3.281 (3)	150 (3)
N51—H51A···Cl1^iii^	0.88 (1)	2.54 (1)	3.396 (3)	165 (3)
N51—H51B···O11	0.88 (1)	2.11 (2)	2.781 (3)	133 (3)
N12—H12···O2M^iv^	0.87 (1)	2.14 (2)	2.912 (3)	149 (3)
N14—H14A···Cl1^v^	0.88 (1)	2.34 (1)	3.188 (3)	162 (3)
N14—H14B···O2M^iv^	0.88 (1)	1.93 (2)	2.783 (3)	162 (3)
N15—H15A···Cl1^v^	0.88 (1)	2.61 (2)	3.367 (3)	145 (3)
N15—H15B···O11	0.88 (1)	2.03 (3)	2.688 (3)	131 (3)
O1M—H1M···Cl1	0.84	2.26	3.091 (2)	171
O2M—H2M···O1M	0.84	1.91	2.745 (3)	170

Symmetry codes: (i) −*x*+2, −*y*, −*z*+1; (ii) −*x*+3/2, *y*−1/2, −*z*+1/2; (iii) *x*+1/2, −*y*+1/2, *z*+1/2; (iv) −*x*+1/2, *y*−1/2, −*z*+1/2; (v) *x*−1, *y*, *z*.
